# Dynamic of land use and vegetation change in the eastern bank of Bénoué (North Cameroon)

**DOI:** 10.1186/s40529-024-00413-3

**Published:** 2024-03-13

**Authors:** Djosebe Azaria, Froumsia Moksia, Tchobsala, Kamblaba Pierre, Prudence Tezore Bakary

**Affiliations:** Department of Biological Sciences, Faculty of Science, University of Maroua, P.O Box 814, Maroua, Cameroon

**Keywords:** Dynamic of land use, Vegetation change, East bank of the Bénoué, Cameroon

## Abstract

The eastern part of the Benoue River bank is undergoing degradation marked by a significant decrease in vegetation cover and woody resources due to anthropogenic activities and climatic. The main objective of this study is to analyze the farmers’ knowledge of vegetation evolution and the dynamics of land use using satellite images in the east of the bank of the Benoue. The methodological approach used is an integrated one combining field surveys, remote sensing, mapping, and modeling. The results obtained show that 88% of the population surveyed believe that the area covered by vegetation has decreased. The reasons for this decrease are numerous, but the main one remains the strong anthropic activity that would be at the origin of the progressive degradation of the land. The evolutionary trend of plant formations is essentially regressive for natural formations from 1991 to 2021. The analysis of the evolution of land use showed that in the Rey-Bouba district during 1991, 58.24% of the area formerly made up of dense woody formations regressed considerably to 25.77% in 2021. The same is true for the Bibemi district where the area of wooded zone has decreased from 65.47% in 1991 to 28.45% of the total area in 2021. This regression of the surface area of wooded formations was done to the benefit of anthropized occupation classes whose area has increased. They suggest an effective awareness in the monitoring of the dynamics of the vegetation cover subjected to anthropic pressures and climatic variations for a better-integrated management of the vegetation of this area.

## Introduction

The expansion of agricultural areas linked to population growth is one of the main drivers of forest cover degradation in the world. This situation is particularly visible in developing countries where forest areas are subject to numerous pressures due to poor natural resource management (Boussougou et al., 2017). Africa’s forests are experiencing rapid processes of rural landscape transformation and forest resource degradation (Ariori and Ozer 2005; Larwamou et al*.,* 2005; Mama et al. 2013). Between 1900 and 2000, the FAO estimated that the African continent lost 52 million hectares of forest, accounting for 56% of the global forest cover reduction (FAO 2014). This degradation of natural resources is mainly attributable to unsustainable management practices (deforestation, overgrazing, high and ever-increasing demographics, growing energy needs, overexploitation of fodder resources, increased industrial exploitation) (Abdourhamane et al. 2012; Ballo et al. 2016; Temgoua et al. 2018a). In Cameroon, pressure on land and natural resources appears to be increasing, whether for subsistence, firewood, grazing, or logging needs (Youssaou 2011). Currently, in terms of land use, the forest has retreated by 619 km^2^ and cultivated land has increased by 321 km^2^. The surface area of degraded forests and land is estimated at approximately 12 million hectares with a general tendency to worsen the phenomenon due to both natural and anthropogenic factors (MINEPDED 2017). Agricultural colonization has as a corollary the dynamics of land use. Understanding the mechanisms underlying the evolution of natural resources and optimizing their role requires taking into account the insertion of these resources in land use planning. Remote sensing and mapping offer an immense source of data to study the spatial and temporal dynamics of environmental parameters. They can provide timely synoptic information for the identification and monitoring of local territories (Smith 2012). In addition, they play a great role in applications such as environmental damage assessment, land use monitoring, urban planning, soil and crop yield assessment (Avakoudjo et al. 2014). Thematic maps illustrating the topography and land use systems can be distinguished by interpretation of satellite imagery, using trends and conversions. Detection of LUCC using geospatial techniques is useful in supporting government policies for efficient utilization of land (Sarfo et al. 2022). The consequences of the pressures exerted on vegetation for decades have led many researchers to take an interest in the problem of biodiversity management. In this context of strong anthropic pressure and climate change, it is urgent to evaluate the dynamics of plant communities. The general objective of this study is to analyze the farmers' knowledge of the evolution and dynamics satellite images in the eastern part of the Benoue river bank in view of a sustainable management of the vegetation cover in this area. More specifically, the project aims to: (i) Evaluate farmers’ perception of the evolution of vegetation and the factors which influence this evolution, (ii) Produce image material highlighting the qualitative evolution of changes in vegetation cover, (iii) Identify quantitative descriptors of the spatial distribution of vegetation cover in this area. This study is based on the hypothesis that the expansion of cultivated areas in the zone combined with the effects of unsustainable management practices are involved in the spatio-temporal dynamics of the eastern bank of the Benoue.

## Materials and methods

### Study site

#### The district of Bibemi

The Bibemi district is located in the Benoué department in the North region. It shares its southern boundary with the district of Lagdo, to the north with the Department of Mayo-Louti, to the east with Tchad and Mayo Rey and to the west with the district of Pitoa. The Bibemi district covers an area of 2535 km^2^. Bibemi has an estimated population of 236,000. It is made up of the following ethnic groups: Foulbé, Moundang, Mambay, Guiziga, Toupouri, Kapsiki, Arab, Haoussa, Bornowa, Kâ-Ngou, Lamé, Fali, Gambay, Laka, Kera, Massa, Guidar, Mada, Matal, Mafa and Zoulgo. The population of the Bibémi district practices two religions: Islam and Christianity. (PDC commune de Bibemi 2014) The population of Bibémi lives mainly from agriculture and livestock farming. The climate is tropical, of the Sudano-Sahelian type, with a long dry season from October to April and a short rainy season from May to September. Average annual rainfall reaches 956 mm of water in 54 days of rainfall (MINADER, 2014). Temperatures remain high, with an average of 31 °C, and maximums reaching 42–45 °C in April. Predominantly ferruginous, soil diversity is a characteristic feature of the soils in the district.

#### The district of Rey-Bouba

The district of Rey-Bouba is located in the North Cameroon Region, Mayo-Rey Department. It borders Tchad to the east, the district of Lagdo to the west, the district of Madingrin and Tcholliré to the south and the district of Bibémi to the north. It was created in 1966 and covers an area of 8000 km^2^. It has a population of nearly 100,000. The main ethnic groups living in this commune are the Peulhs, Damas, Lélés, Toupouri, Dourous, Moundang and Gambaye (PDC commune de Rey-Bouba 2011). The climate is Sudanian. The year is divided into two main seasons of equal length. The rainy season runs from April to October. Annual rainfall is around 900–1300 mm. Rainfall is irregular and concentrated mainly around August. The dry season extends from November to March. Thermal variations are significant. Temperatures are around 28 °C in the rainy season, 17 °C in the cool season and rise to around 40 °C in the hot season. The terrain is very rugged. It is made up of mountains, the most important of which is Taparé. It is the highest peak in the region. This relief is conducive to the formation of low-lying areas that are ideal for farming. The district is criss-crossed by a number of rivers, the most important of which is the Mayo-Rey, which runs almost the entire length of the district. Agriculture and livestock farming are the main activities in the area.

### Methodology

The methodological approach used in this study based on field surveys, remote sensing, and the geographic information system (GIS).

#### Field surveys

A questionnaire was administered to the population in 20 villages in the area, including 10 villages in the Bibemi district and 10 villages in the Rey-Bouba district. These villages were selected on the basis of their accessibility, the practice of vegetation harvesting and logging activities. The stratified probability sampling method (sex, age) described by (Grangé and Lebart l992) was adopted. We opted for these two quotas not only because perceptions vary with sex and age, but also because people aged 30 or older are the most likely to give us information by asking their memories. We surveyed 165 people in the Bibemi district and 163 people in the Rey-Bouba district, for a total of 328 people. It was a question of tracing the history of the occupation of the sites by the populations by giving landmark dates. In order to do this, all people who were at least 30 years old and native to the area could be surveyed. Men, women, traditional authorities, notables and elderly people were taken into account.

#### Mapping

For the mapping, we used a Global Positioning System (GPS) to locate the 20 villages selected in the two districts of our study area. This allowed us to map and characterize the different vegetation formations in the area. Other types of data, such as those from the SOGEFI 2019 database, have been used to represent rivers, roads, administrative subdivisions, lakes.

#### Field observation and photography

The direct observation in the wild and in some target villages allowed to evaluate the conditions in which the vegetation evolves in this area and to identify the problems.

#### Satellite data

The achievement of the objectives related to the land cover map implies a judicious choice of satellite images. Not only did we have to cover almost the entire study area, but we also had to choose the appropriate dates that fit with the realities of the sites and also take into account the availability of satellite images. The period selected for image acquisition corresponds to the dry season. Jensen (1983) recommends this period for studies aiming at detecting observable changes in vegetation. He argues that images acquired during dry periods for change detection have high contrast and reduce problems related to differences in solar angles, dissimilarities in soil moisture, and phenological changes in vegetation. During this period, vegetation cover and chlorophyll activity are still discernible and the presence of bare space is also distinguishable. Cloudiness, one of the factors that can alter the quality of images is also reduced. The inter -annual rainfall variation exerting a great influence on the vegetation is another relevant criterion for image selection (Diallo et al*.,* 2011). In the present work, three satellite images dating from 1991, 2006 and 2021, were analyzed (Table 1).

**Table 1:** satellite and its characteristics

Images	Sensors	Acquisition periods	Number of bands	Resolution (m)	Season
LANDSAT 4,7	MSS, ETM +	1991 2006	8	30 m	Dryer
Landsat 8	OLI and TIRS	2021	11	30 m	Dryer

### Evaluation of the dynamics of land use

- ❖ Preprocessing of satellite images

The channels from visible to near infrared were used for this study, because of the best information they provide on land use (Essifi. 2008). Then to adjust the degree of disturbance of these different channels, radiometric corrections were made. In addition, a colored composition (false color) was used to obtain the best image quality visualization of the image objects (Sarr 2009).

- ❖ Digital classification of the images

The vegetation type was identified according to the Yangambi typologịe established for the mapping of vegetation (Aubreville 1957). Given the knowledge of the terrain, the work was based on digitization and visual classification on the screen, a technique that consists of manually and exhaustively digitizing all the homogeneous units of the false color images of the study area and the color images of the study area and to proceed to their classification.

- ❖ Post-classification processing and validation of results

**Fig. 1:**
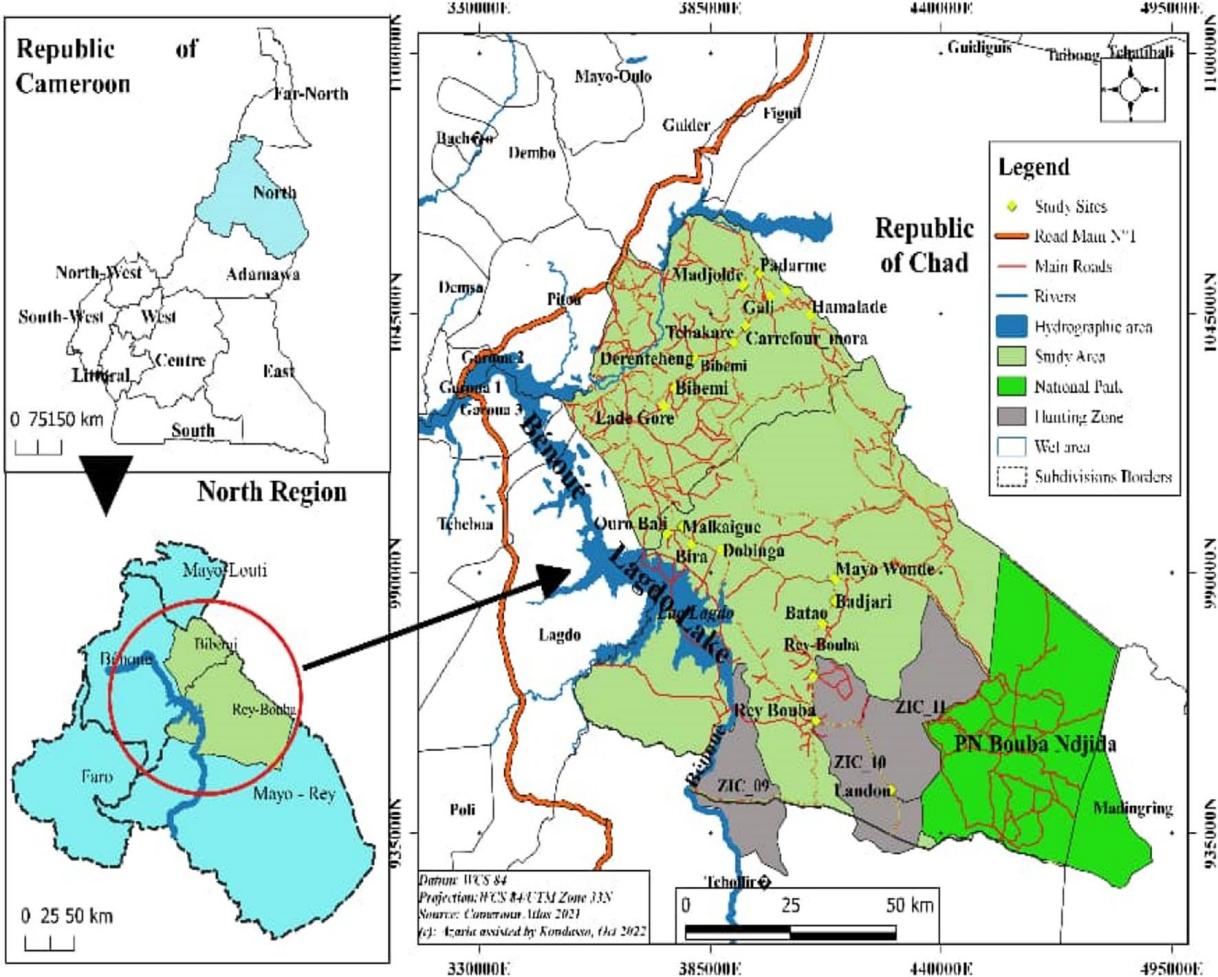
Location map of the study area

Confusion matrices were generated after classification of the selected scenes. The omission and commission errors were calculated for each land cover unit and the values obtained indicate the accuracy of the interpretation of each class. Finally, the total accuracy is given by the Kappa (Pontius 2000). This index incorporates all the elements of the confusion matrix and is widely used in the evaluation of the accuracy of a satellite image classification and in change detection methods (Foody 1992). K > 0.80 represents strong agreement and good accuracy; K between 0.40 and 0.80 means a middle accuracy, and K < 0.40 represents a poor accuracy (Obahoundje et al. 2017; Akpoti et al. 2016). The kappa coefficient is given by the Eq. (1).

1 $$ K\, = \,\frac{{N\sum\limits_{i = 1}^{r} {x_{ij} \, - \,\sum\limits_{i = 1}^{r} {\left( {x_{i + } \,*\,x_{ + i} } \right)} } }}{{N^{2} \, - \,\sum\limits_{i = 1}^{r} {\left( {x_{i + } \,*\,x_{ + i} } \right)} }} $$

where r is the number of rows in the matrix, xij is the number of observations in row i and column i, xi + and x + i are the marginal totals of row i and column i, respectively, and N is the total number of observations (Fig. 1).

### Statistical analysis of quantifications of change in land cover classes

In order to quantify the changes in land use classes, several statistical indicators were calculated. These are:

#### Annual rate of change

This index was calculated to appreciate the evolution of the different occupation units from 1991, 2006, and 2021 and to determine the different orders of regression (1, 2, 3,…n), progression (1, 2, 3, …n) and stability because the dynamics is a reflection of the effects of the population activities on the vegetation cover. Thus by means of the formula proposed by FAO (1995) and whose use is very frequent (Tsewoue et al. 2020), the rate of change Tv (%) of land cover areas is calculated according to the formula:

2 $$\mathbf{T}\mathbf{v}(\mathbf{\%})=\frac{{\varvec{S}}2-{\varvec{S}}1}{{\varvec{S}}1}x100$$

With S1 the area of a land use category at date t1; S2 the area of the same land use category at date T2, with 2 > 1.

- S2-S1 = 0: there is stability (S) of the vegetation cover from year 1 to 2;
- S2-S1 > 0: there is progression (P) of the vegetation cover from year 1 to 2;
- S2-S1 < 0: There is regression of vegetation cover from year 1 to 2.

#### Average annual rates of spatial expansion

This expresses the proportion of each land cover category that changes annually. From the area of each land cover category, this rate has been calculate according to the formula of Bernier (1992):

3 $$\mathbf{T}\mathbf{c}=\frac{lnS2-lnS1}{\left(t2-t1\right)*lne}x100$$

S1 and S2 the area of a landscape unit at date t1 and t2 respectively; t2-t1 the number of years of evolution; ln the neperian logarithm, e the base of the neperian logarithm (e = 2.71828).

#### Speed of evolution of the categories of land use

It is calculate according to the formula:

4 $${\varvec{\Delta}}\mathbf{S}=\frac{S2-S1}{t2-t1}$$

ΔS: speed of change (extension or regression in ha/year).

## Results

### Farmers' perception of the evolution of vegetation over the years

**Fig. 2:**
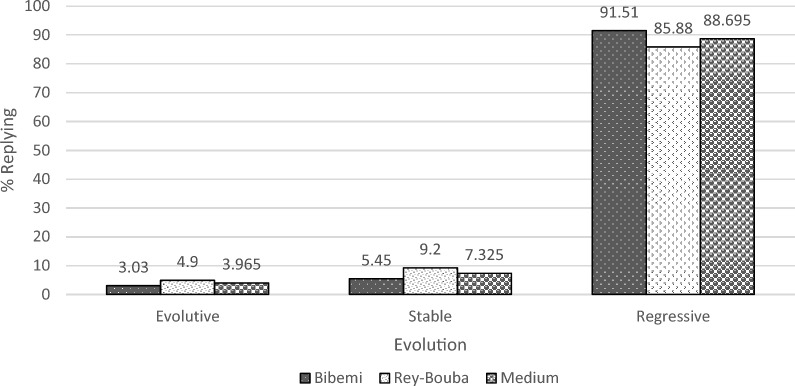
Farmers perception of the evolution of vegetation over the years

Analysis of Fig. 2 indicates the assumption of the vegetation in the eastern part of Benoue regressing over the years in terms of surface area are the most represented (88.69%) compared to those who assume its stability (7.32%) or increment (3.96%). This way of perceiving the evolution of the vegetation in this area varies in terms of percentages of respondents from one district to another. This is why in the Bibemi district; the local populations think that the area of vegetation in this locality is decreasing over the years. Indeed, the people of this district point out that the gallery forests and savannahs are cutting down each year to the benefit of farmlands. In the sub-division of Rey-Bouba, the people living in this locality assume that the evolution of vegetation in this area over the years has been more regressive (85.88%). The latter believes aside influential factors like agriculture and livestock/grazing, the excessive or unregulated cutting of wood and poor forest control would lead to a gradual decline in vegetation over time. Few believe that the vegetation in this area is stable (9.2%) or increasing (4.9%).

### Degradation activities and vegetation cover dynamics

**Fig. 3:**
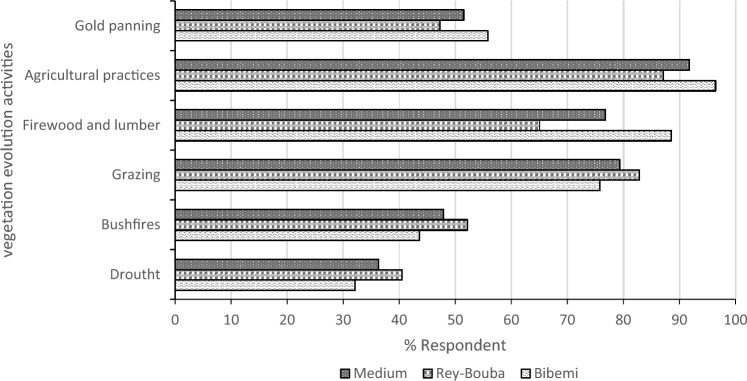
Ranking of causes of resource reduction

**Fig. 4:**
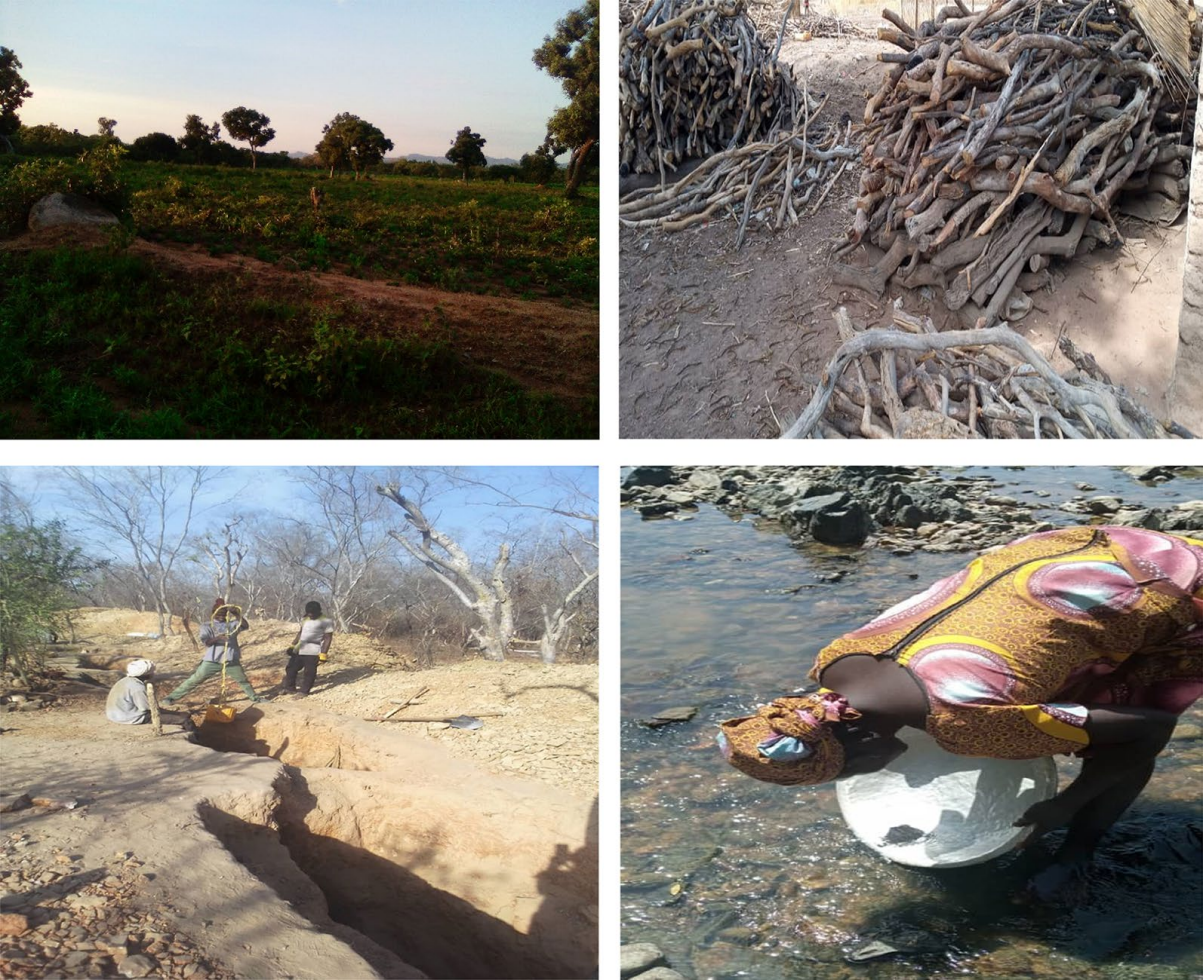
Degradation activities and vegetation dynamics

Eighty-eight percent (88%) of the surveyed population believe that the area covered by vegetation in this zone has considerably decreased. The reasons for this progressive decrease are numerous. Currently, the activities that are responsible for the dynamics of the vegetation are, among others, the practice of agriculture, the exploitation of firewood and timber, overgrazing, bush fires, gold processing, and droughts. Agriculture is the main activity that primarily degrades natural plant formations in this area, according to 91.73% of respondents. This activity is followed by grazing and the harvesting of firewood and timber, covering 79.28 and 76.75% of respondents respectively (Fig. 3). These could be attributed to the population in the area largely being dependent on agriculture; hence, the increasing demand for agricultural land and reliance on wood for energy for domestic purposes (Fig. 4). Similarly, droughts or prolonged dryness influenced land cover change in the study domain.

### Evolutionary dynamics of vegetation cover

#### Validation of the 1991, 2006 and 2021 LANDSAT image classifications

The result of the classifications of the LANDSAT images allowed us to identify eight land cover classes. Table 2 shows the Kappa index calculated by rounding and by the image/data acquisition year. Thus, for the 1991, 2006 and 2021 LANDSAT imagery classifications, the kappa coefficient was respectively 0.98 (98.61% global accuracy), 0.91% (91% global accuracy) and 0.99 (99.74% global accuracy) for the images of the Rey-Bouba district. Classification for LANDSAT images for 1991, 2006 and 2021 of the Bibemi area, based on the kappa coefficient generated 1.00 (100% global accuracy) for 1991, 2006 and 0.99 (99.38% global accuracy) respectively. The kappa coefficients improve as we evolve in time. Indeed, we notice that the 2021 classification presents less confused classes than the two other classifications. Overall, the kappa coefficient and the accuracy of the LANDSAT image classifications are better.

**Table 2:** Evaluation of the accuracy of land cover maps

Bibemi			Rey-Bouba		Evaluation of Kappa Index
Land cover years	Kappa coefficient (K)	Overall accuracy (%)	Kappa coefficient (K)	Overall accuracy (%)	
1991	1.00	100	0.98	98.61	K > 0.81 Excellent 0.61 < k < 0.8 Good 0.41 < k < 0.6 Moderate 0.21 < k < 0.4 Poor
2006	1.00	100	88.55	91	
2021	0.99	99.38	0.99	99.74	

#### Evolution of vegetation cover in the eastern part of the Benoue River bank

The analysis of the evolution of the vegetation cover in the eastern part of the Benue River bank was highlighted by the analysis of satellite images separately in two districts covering this area.

#### Status of land cover in 1991, 2006, 2021 in the Rey-Bouba district

**Fig. 5:**
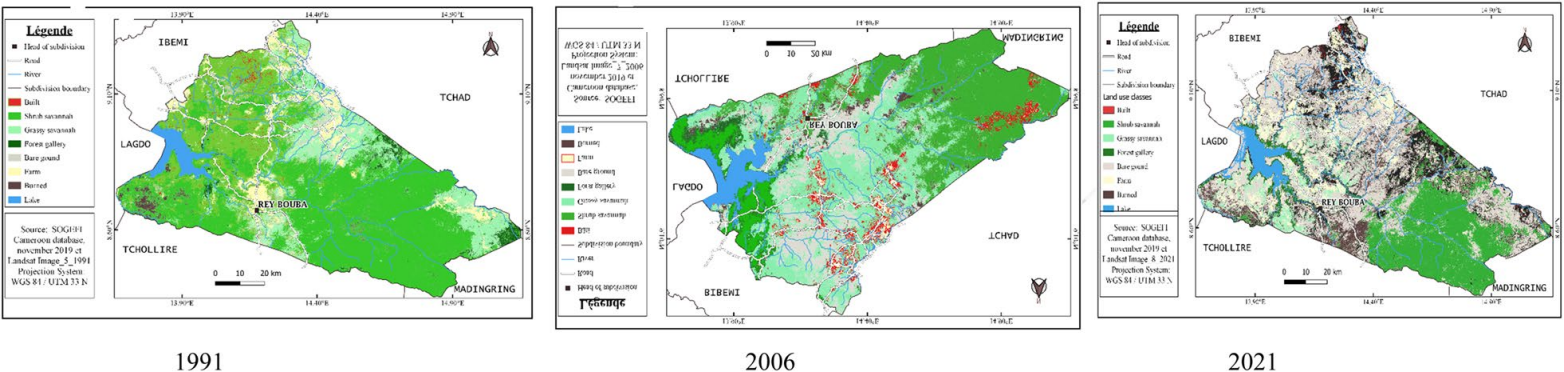
Land use map of 1991, 2006, and 2021 of the Rey-B site from the lansat image

**Fig. 6:**
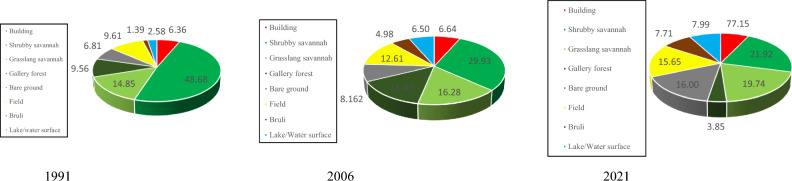
Percentage of occupancy units 1991, 2006 and 2021 for the Rey-Bouba site

The status of land cover and rural space in the Rey-Bouba area for the years 1991, 2006, 2021 is summarized in Figs. 5 and 6. Eight land cover classes were mapped in this district. These land cover classes vary from year to year and from class to class. The cartographic result of the 1991 image and the percentage of occupation of each class shows that the “shrubby savannah” class is the most represented because it occupies 48.60% of the total surface of the area. This type of formation is located almost on the entire surface of this study area. The «grassy savannah» class (14.85%) follows it. This class is located inland and to the east of the Rey-Bouba district. The farmlands represent 9.71% of the total area of the zone and is the third occupation class that occupies more area. Gallery Forest and bare soil occupy 9.56 and 6.81% of the total area of the site respectively. Buildings cover 6.36% of the total study area. The weakest occupancy classes are observed in the formations Lakes/water surfaces and Burned, occupying respectively, 2.58 and 1.39%, of the total area of the area.

Examination of the different land-use units in 2006 compared with the reference year (1991) shows a decrease in the area, shrubby savannah (29.93%), other times occupying 48.60% to the benefit of the other occupation classes Grassy Savanna (16.68%), Gallery Forest (14.47%), Field (12.61%), Bare Ground (8.16%), Built-up (6.64%), Lakes/Surfaces (6.5%) other times occupying respectively 9.56, 9.71, and 6.81, 6.36, and 2.58% of the total surface of the site. As for gallery forest, the increase in its surface area may be due to the stability of the environment, which would have facilitated the reconstitution of vegetation in this occupation class. Illustration of the proportions in percentage and map of land use units in the year 2021 in the district of Rey-Bouba shows an increase in the surface area of the field occupancy from 9.71% of the total area in 1991 to 15.65% of the total area of the zone in 2021. It can be seeing that since 1991 the area of this occupation class is increasing. The increase in the surface area of the farmland’s occupation class could be due to the growing need for arable land of the population living in this area. It has the effect of converting the other most represented occupation classes such as shrubby savannah whose occupation surface has dropped to 21.92% in 2021. The grassy savannah occupies 19.75% of the total area of the zone. They cover a large area compared to previous dates. Bare soil occupies an area of 16%. The surface of lakes/water surfaces covers more than 7.99% of the study area. Built-up areas account for 7.15% of the total area, a slight increase in area from 1991 and 2006. The gallery forest and burnt forest classes account for 3.85% and 7.71% of the total area of the study area respectively.

#### Status of land cover in 1991, 2006, 2021 of Bibemi District

The cartographic result and the percentage of land use units vary from one land use unit to another and from one year to another. The analysis of the 1991 land use map allows us to distinguish the different land use classes such as shrub savannah, grassy savannah, built-up areas, fields, bare soil, water surface, and burned land in this area. The “shrub savanna” class is the most represented. It occupies nearly 51.90% of the study area. It is followed by the “Bare ground gallery forest” class, which occupies 13.53% of the surface area of the study area. The class of burning occupies an area of 11.03%. Its three classes of occupation cover alone 76.46% of the total area of the area. The water surface, field, buildings, bare soil, and grassy savannah are the occupation classes that were poorly represented in 1991. They cover respectively 6.00, 5.39, 5.00, 4.79, and 2.33% of the total area of the zone.

A closer look at the different land-use classes in 2006 shows that shrub savanna is heavily occupied, accounting for 46.01% of the total area of the zone. We note a decrease in surface area of 5.89% of this class of occupation compared to the year 1991. Followed by the class of gallery forest occupying 10.87% of the area. The water surface occupies 8.58% of the total area of this zone. The burned and the farmland occupy respectively 7.97 and 7.32% of the total area of the study area. The low percentage of occupation is observed in the classes of grassy savannah, built and bare soil occupying respectively 6.48, 6.31 and 6.45% of the total area of this area.

**Fig. 7:**
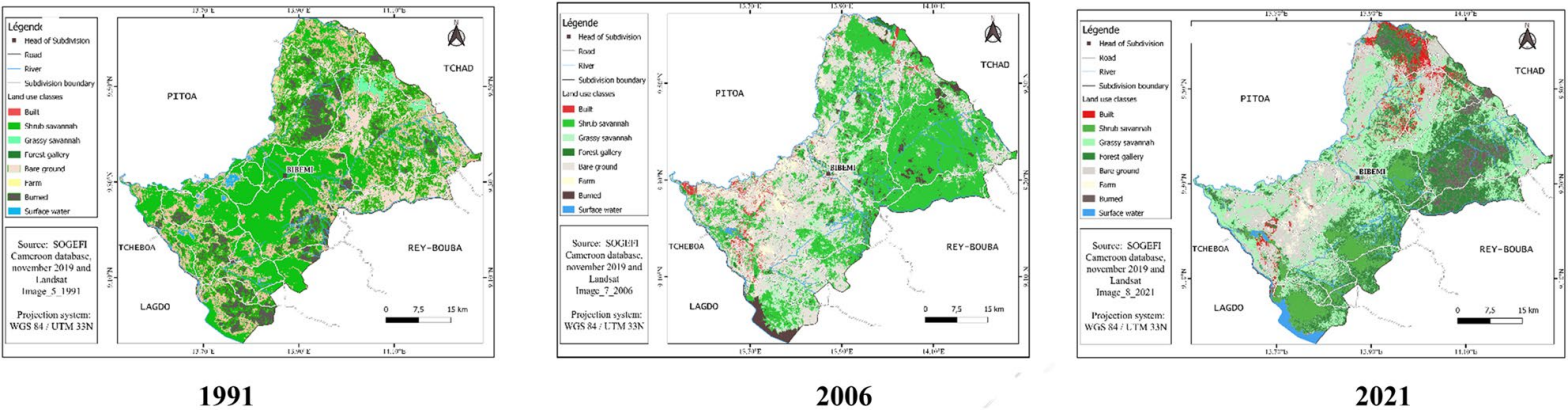
Land use map of 1991, 2006, and 2021 of the Bibemi site from the lansat image

**Fig. 8:**
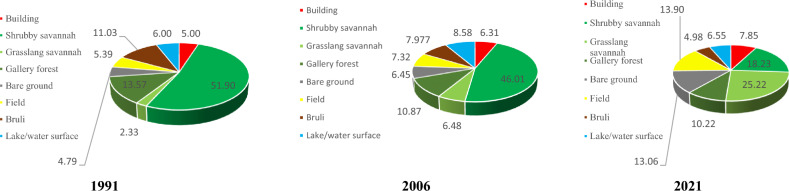
Percentage of occupancy units 1991, 2006 and 2021 for the Bibemi site

**Fig. 9:**
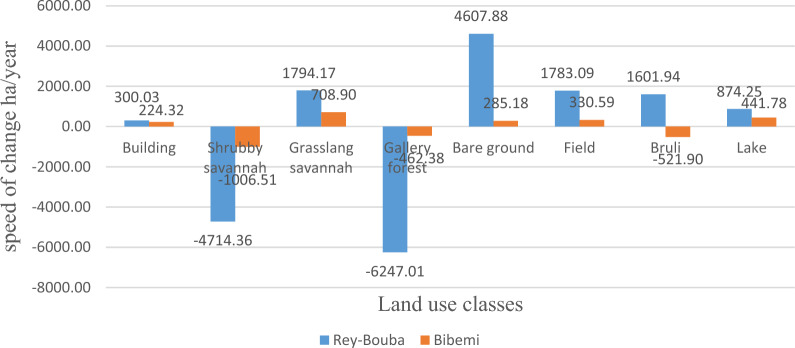
Rate of land use change between 1991 and 2006

As for the cartographic result of the 2021 image (Fig. 7), the share of each land use class is highlighted by the percentage occupied by each of them (Fig. 8). The “Grassland Savanna” class is the most represented, occupying 25.22% of the total area. It can be seen that this occupation class has an increasing surface area since 1991, thus justifying the influence of anthropic activities in this area. It is followed by the shrub savanna class which occupies 18.23% of the total area of the zone. This class of occupation has considerably decreased in area to the benefit of other anthropogenic classes. The farmlang is the third most represented land use class in 2021 in this area. It occupies 13.90% of the total area of this zone. The “bare soil” and “gallery forest” occupy respectively 13.06% and 10.22% of the total area of this zone. Its occupation classes are located both inside and outside this area. The other classes of occupations are weakly represented. These are the built-up areas, water surface, and burned areas occupying respectively 7.85, 6.55, and 4.98% of the study area (Fig. 9).

The regression of shrub savannah and gallery forest in this area would be due to anthropogenic actions favoring the transformation of shrub savannah, gallery forest, into other land use classes.

### Spatial and temporal changes in land use

#### Detection of spatio-temporal changes between 1991 and 2006

In order to understand the changes that have occurred and to identify the causal forces that explain them, more detailed observations were made on each of the land use units. Examination of the annual rates of change, the rate of expansion (Table 3) of the different land use classes shows that the changes that have occurred vary from one land use unit to another. Some land uses have increased in size while others have decreased in size in the two districts covered by the area. In the district of Rey-Bouba, shrubby savannahs have lost their surface area (− 165283.74/ha/year, i.e., a rate of decrease of − 38.51%/year) in favor of other classes. The classes of built-up areas, grassy savannah, gallery forest, bare ground, farmland, burnt areas and lakes have seen their areas increase with a rate of change of 4.29; 12.32; 51.21; 19.69; 29.89; 256.4% and 151.68% respectively The average annual rate of expansion of the Brulis is estimated at 8.45% corresponding to an area of 31612.68 ha/year, the lake/water surface has an expansion rate of 6.13% equivalent to an area of 34546.61 ha/year, the grassy savannah has an estimated expansion rate of 0.77% covering an area of increase of 16132.62 ha/year in this area. The gallery forest, bare ground, farmland, and built-up areas cover respectively an expansion rate of 2.75, 1.19; 1.73; 0.28%; that is to say an area of increase of 43217.91 ha; 11834.5 ha/year, 25529.49 ha/year and 2409.93 ha/year. The same is true for the land use classes in Bibemi district with a progressive trend for the Built-up, Grassy Savannah, Bare Ground, Farmland, and Water Surface classes with an evolution rate of 26.26, 177.62, 34.86, 35.89 and 43.08% respectively. The average annual rate of expansion is 1.55% corresponding to 3364.85 ha/year for the Built, 6.79% equivalent to 10633.56 ha/year for the grassy savannah, 1.99% or 4277.73 ha/year for the bare soil, 2.04% corresponding to 4958.91 ha/year for the Farmland and 2.38% for 6626.7 ha/year water surface. The regressive trend was observed in the occupation class, the shrubby Savannah, gallery forest, and bushland with respectively for annual rate of decrease estimated at − 11.34, − 19.93 and − 27.68%. The regressive trend noted in the shrub savannah, gallery forest, and scrubland class can be explained mainly by the exploitation of plant species found in this plant formation, overgrazing and the expansion of cultivable areas.

**Table 3:** Rate of change of land use units within the same class (period 1991–2006)

	Occupancy classes	Area (ha) 2006	Area (ha) 2021	Change (2021–2006) ha	Rate of change	Expansion rate
	Building	56129.76	58539.69	2409.93	4.29	0.28
	Shrubby savannah	429190.83	263907.09	− 165283.7	− 38.51	− 3.23
	Grasslang savannah	130936.5	147069.12	16132.62	12.32	0.77
Rey-Bouba	Gallery forest	84388.68	127606.59	43217.91	51.21	2.75
	Bare ground	60115.68	71950.18	11834.5	19.69	1.19
	Farmlang	85676.31	111205.8	25529.49	29.89	1.73
	Bruli	12328.83	43941.51	31612.68	256.41	8.45
	Lake/Water surface	22775.94	57322.55	34546.61	151.68	6.13
	**Total**	**881542.53**	**881542.53**			
	Building	12813.12	16177.973	3364.85	26.26	1.55
	Shrubby savannah	133086.06	117988.35	− 15097.70	− 11.34	− 0.8
Bibemi	Grasslang savannah	5986.71	16620.265	10633.56	177.62	6.79
	Gallery forest	34803.405	27867.78	− 6935.62	− 19.93	− 1.48
	Bare ground	12271.32	16549.05	4277.73	34.86	1.99
	Farmland	13815.9	18774.81	4958.91	35.89	2.04
	Bruli	28277.505	20449.08	− 7828.42	− 27.68	− 2.15
	Lake/Water surface	15382.44	22009.14	6626.7	43.08	2.38
	**Total**	**256436.46**	**256436.46**			

#### Spatial and temporal change detection between 2006 and 2021

The area of land use units between 2006 and 2021 shows a variation between land use classes. It can be seen that some land use units have increased in area while others have decreased in area. The statistical analysis in Table 4 shows that in the Rey-Bouba district, the area of bare land, burnt land, farmland, lake, grassy savannah and built-up areas have increased with an evolution rate of 96.06, 54.68, 24.05, 22.88, 18.3 and 7.69% respectively, with an annual expansion rate of 4.47, 2.9, 1.43, 1.37, 1.12 and 0.49%. This increase is done at the rate of 69118.19 ha/year for bare soil, 24029.1 ha/year for burnt land, 26746.38 ha/year for farmlang, 13113.76 ha/year for lake/water surface, 26912.58 ha/year for grassy savannah and 4500.45 ha/year of built-up areas. On the other hand, the area of shrub savannah and gallery forest decreased significantly between 2006 and 2021 with a decrease rate of − 26.8 and − 73.43% respectively for an annual expansion rate of − 2.07 and 8.81%. This loss is estimated respectively at − 70715.34 ha/year for shrub savannahs and − 93705.12 ha/year for gallery forests. As for the changes between the classes of occupations in the Bibemi district, it should be noted that the class of grassy savannahs, bare soil, farmlands, and buildings have increased in surface area for a respective rate of change of 289.08, 102.42, 89.89, and 24.35% for an annual rate of expansion of 9.03, 4.69, 4.26, and 1.45%. This change in the area of the different land use classes reflects the vegetation dynamics that this area undergoes between these two dates.

**Table 4:** Rate of change of land use units within the same class (period 2006–2021)

	Occupancy classes	Area (ha) 2006	Area (ha) 2021	Change (2021–2006) ha	Rate of change	Expansion rate
	Building	58539.69	63040.14	4500.45	7.69	0.49
	Shrubby savannah	263907.09	193191.75	− 70715.34	− 26.8	− 2.07
	Grassland savannah	147069.12	173981.7	26912.58	18.3	1.12
Rey-Bouba	Gallery forest	127606.59	33901.47	− 93705.12	− 73.43	− 8.81
	Bare ground	71950.18	141068.36	69118.19	96.06	4.47
	Farmland	111205.8	137952.18	26746.38	24.05	1.43
	Bruli	43941.51	67970.61	24029.1	54.68	2.9
	Lake/Water surface	57322.55	70436.314	13113.76	22.88	1.37
	**Total**	**881542.53**	**881542.53**			
	Building	16177.97	20117.28	3939.55	24.35	1.45
	Shrubby savannah	117988.35	46736.98	− 71251.56	− 60.39	− 6.15
	Grassland savannah	16620.26	64665.45	48045.18	289.08	9.03
Bibemi	Gallery forest	27867.78	26204.62	− 1663.16	− 5.97	− 0.41
	Bare ground	16549.57	33498.45	16949.29	102.42	4.69
	Farmland	18774.81	35651.19	16876.31	89.89	4.26
	Bruli	20449.08	12759.84	− 7689.24	− 37.6	− 3.13
	Lake/Water surface	22009.14	16802.46	− 5206.68	− 23.66	− 1.79
	**Total**	**256436.46**	**256436.46**			

### Rates of land use change

#### Speed of land use changes between 1991 and 2006

The speed of land use changes between 1991 and 2006 shows a great difference between the different land use classes. We can thus observe the decrease of some land use classes while others have increased. Figure 6 shows the rate of change of land use between 1991 and 2006 in two districts of this zone. The figure shows that in the Rey-Bouba district, two (02) land use classes (shrub-savanna and gallery forest) of the eight (08) land use units have lost their surface area by − 4714.36 ha/year and − 6247.01 ha/year respectively. This loss of land area is also observed in the Bibemi district for the shrub savanna (− 1006.51 ha/year), gallery forest (− 462.38 ha/year), and burnt land (− 521.90 ha/year). As for the other classes of occupations, grassy savannah, bare ground, farmland, burnt land, lake and built-up areas, they have increased in terms of surface area at an increasing rate of 1794.17 ha/year; 4607.88 ha/year; 1783.09 ha/year; 1601.94 ha/year; 874.25 ha/year; and 300.03 ha/year for the Rey-Bouba district. In Bibemi district, this increase in the rate of land use change was observed for the savanna grassland (708.90 ha/year), bare soil (285.18 ha/year), farmland (330.50 ha/year), lake/water surface (441.78 ha/year), and built-up (224.32 ha/year) land use classes. The large variation in rates of change observed between the different land use units in this area reflects the high pressure of human activities on the natural environment in this area.

#### Rates of land use change between 2006 and 2021

**Fig. 10:**
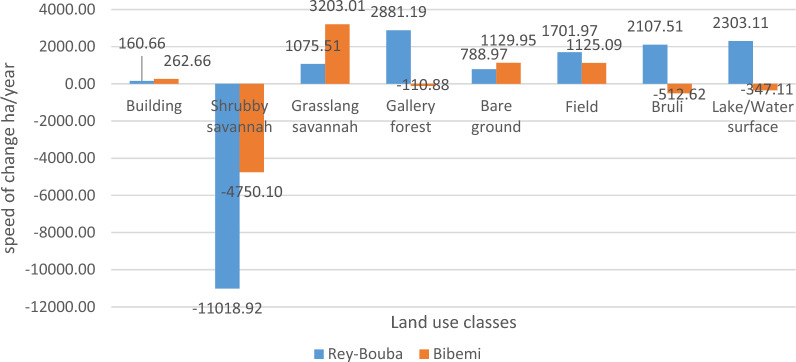
Rate of land use change between 2006 and 2021

The speed of land use change shows that between 2006 and 2021 the shrub savannah surfaces has a loss speed of − 11018.9210 ha/year for the Rey-Bouba district and − 4750.10 ha/year, − 110.88 ha/year; − 512.62 ha/year, and − 347.11 ha/year respectively for the shrub savannah, gallery forest, bushland and lake/water surface in the Bibemi district. However, the classes of built-up areas, grassy savannah, gallery forest, bare soil, bush, farmland, bush, and lake have seen an increase in their surface areas in the Rey-Bouba district, with a rate of change of 160.66 ha/year, 1075.51 ha/year, 2881.19 ha/year, 788.97 ha/year, 1701.97 ha/year, 2107.51 ha/year, and 2303.11 ha/year respectively. In Bibemi, this increase was observed in the following land use classes: buildings (262.66 ha/year), grassy savanna (3203.01 ha/year), bare soil (1129.95 ha/year) and farmland (1125.09 ha/year) (Fig. 10). This result is undoubtedly related to the expansion of cultivated areas, which generates an increasingly important pressure on wooded areas, thus favoring the expansion of anthropized land use units. Another explanation is the creation of new villages due to the strong migration of populations to the area for mining and the search for better living conditions.

#### Summary of the evolution of the different land use units from 1991 to 2021

**Fig. 11:**
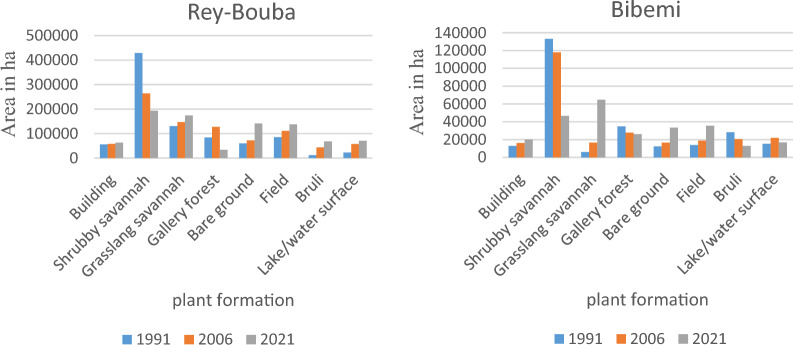
Summary of the evolution of the different land use units from 1991 to 2021

From 1991 to 2021, no natural formations have advanced (Fig. 11). The evaluation of the spatio-temporal dynamics of vegetation in the eastern part of the Bénoue riverbank revealed an increase in the area of certain elements such as the built-up area, which increased from 56129.76 ha (1991) to 63040, 14 ha (2021), grassy savanna from 130936.5 ha (1991) to 173981.7012 ha (2021), bare ground from 60115.68 ha (1991) to 141068.3648 ha (2021), field from 85676.31 ha (1991) to 13,795 ha (2021), burned area from 12328.83 ha (1991) to 67970, 61 ha (2021) and lake/water surface 22775.94 ha (1991) to 70436.314 ha (2021) for the district of Rey-Bouba. The same applies to the class of occupation the built that occupied 12813.12 ha (1991) to 20117.8283 ha (2021), Grassland from 5986.71 ha (1991) to 64665.45 ha (2021), Bare ground from 12271.32 ha (1991) to 33498.34 ha (2021), Field from 13815.9 ha (1991) to 35651.11 ha (2021), Water surface from 15382.44 ha (1991) to 16802.46 ha (2021) for Bibemi district. This increase in surface area is explained by the huge increase in population in this area, which has resulted in an increase in the cultivable surface area and the search for new fertile fields. These different practices, has for effect the conversion of the class of natural occupation, in class of anthropized occupation. In addition, there is the artisanal mining in my area. And all this cumulated with the phenomenon of climate change that leads to the regression of other plant formations such as shrub savannah, and gallery forests.

## Discussion

### Degradation activities and vegetation cover dynamics

The main activities that are practiced in this area and that are responsible for the dynamics of the vegetation are agricultural practice, exploitation of firewood and timber, overgrazing, bushfire, gold panning, drought. This result is similar to that obtained by the FAO (2008) when it points out that 90% of deforestation is caused by unsustainable agricultural practices.

### Validation of the 1991, 2006 and 2021 LANDSAT image classifications

The classification of land cover classes resulting from the analysis of landsat images gave an overall accuracy of more than 90%. The kappa coefficients are improving as that we evolve in time. Indeed, we notice that the 2021 classification has fewer confused classes than the other two classifications. Globally, the kappa coefficient and the accuracy of the LANDSAT image classifications are better. Referring to the values of the Kappa index (Kappa > 0.5), we can, according to Pontius (2000), conclude that the results of this analysis are statistically acceptable. When the kappa is greater than 0.81, it is considered excellent; between 0.61 and 0.8 it is good; between 0.41 and 0.6 it is moderate; between 0.21 and 0.4 it is poor and finally when it is negative, then it is very poor (Landis and Koch 1977).

### Land use status of 1991, 2006, 2021 in the eastern part of the Benue river

Referring to the cartographic results, 08 land use units were identified in the two districts that cover the eastern part of the Benue River bank. Analysis of the 1991, 2006 and 2021 land use maps revealed the progressive and regressive evolution of land use units between these three years. The evaluation of the spatio-temporal dynamics of the vegetation in the eastern part of the Benoue riverbank revealed an increase in the area of certain elements such as the built-up area, which has increased from 56129.76 ha (1991) to 63040.14 ha (2021), grassy savanna from 130936.5 ha (1991) to 173981.70 ha (2021), bare ground from 60115.68 ha (1991) to 141068.36 ha (2021), farmland from 85676.31 ha (1991) to 13795 ha (2021), burned area from 12328.83 ha (1991) to 67970.61 ha (2021) and lake/water surface 22775.94 ha (1991) to 70436.314 ha (2021) for the district of Rey-Bouba and similarly for the class of occupation the built that occupied 12813.12 ha (1991) to 20117.8283 ha (2021), Grassland from 5986.71 ha (1991) to 64665.45 ha (2021), Bare ground from 12271.32 ha (1991) to 33498.34 ha (2021), Farmland from 13815.9 ha (1991) to 35651.11 ha (2021), Water surface from 15382.44 ha (1991) to 16802.46 ha (2021) for Bibemi district. This increase in surface area is explained by the huge increase in population in this area, which has resulted in an increase in the cultivable surface area and the search for new fertile fields. These different practices, has for effect the conversion of the class of natural occupation, in class of anthropized occupation. In addition, there is the artisanal mining in the area. All of this, combined with the phenomenon of climate change, is causing the regression of other plant formations such as shrub savannah and gallery forests. His results are similar to those obtained by Sarfo et al. (2022) in Southwestern Ghana who found that there is evidence of expansion in farmlands/shrubs and built-up areas over the given period. Findings based on geo-statistical analysis illustrated drastic increase in farmlands/shrubs (+ 369.81%) and built-up areas (+ 1288.36%) at the expense of a reduction in forested areas (− 82%), water bodies (− 27%) and bare land (− 18.06). Conversely, 73% of experts asserted that there has decline inforest areas in Southwestern Ghana over the past 50 years. These results corroborate those of Adjonou et al. (2010), Abdouraman et al. (2022) who show that the causes of the degradation of vegetation cover are of anthropogenic origin. Arouna et al. (2010) and Tente et al. (2010) also showed that agricultural activities are the major causes of vegetation degradation. Indeed, several authors have reported that the changes in landscape composition in the Sahelo-Sudanian, Sudanian, Sudano-Guinean and Guinean zones are the result of rapid and progressive anthropization manifested by unsustainable agro-sylvo-pastoral practices (Alohou et al. 2016; Illiassou et al. 2015). The climatic factor, by causing a change in the behavior of farmers, constitutes the main factor of degradation (Wafo et al. 2003). The regression of dense and clear forests (natural formations) in favor of anthropogenic formations (industrial plantations and crop fields and inhabited areas) of 0.52% per year. In the same way, the surface area of vegetation formations is regressing in favor of that of anthropic activities, notably fields, fallow land and settlements. In addition, agricultural activities are the major causes of vegetation degradation (Solefack et al. 2018; Tanougong 2019). Tsewoue et al. (2020) showed that regression of natural vegetation formations is accompanied by biodiversity loss and land degradation. Consequently, the pressure of the populations on the vegetation cover leads to the reduction of the forest stand in favor of agricultural spaces. This disappearance of plant formations is the cause of the increase of greenhouse gases in the atmosphere, thus interrupting the ecological balance (Djohy et al. 2016). The modification of climatic parameters in recent years in Cameroon, particularly the decrease in rainfall and the increase in temperature, induces a decrease in the availability of water resources and consequently influences agricultural production and plant formations (Temgoua et al. 2018b).

## Conclusion

In the eastern part of the Benue River bank, where very little information exists on the spatio-temporal dynamics of vegetation cover evolution, the use of satellite imagery has made it possible in this study to highlight the dynamics of land use by processing landsat images from 1991 to 2021. The results from the satellite images allowed to highlight the changes in vegetation related to human activities at a local scale from 1991 to 2021 in the Bibemi and Rey-Bouba area. After a pre-processing phase of the images, the processing was based on an object-oriented classification. The processing of the LANDSAT image allowed us to update the current state of the land cover in this area with 8 land cover classes identified: shrubby vegetation, grassy savannah, gallery forest, bare soil, field, built-up areas and water surface. In this zone, only the locality of Rey still retains almost half of its vegetation surface. In contrast, the Bibemi area contains only 25% of forest in its surface. The dynamics of land use was highlighted by the land use maps of 1991, 2006 and 2021 made from LANDSAT images. Thus, it was shown that the hypothesis of increased vulnerability to human pressures in this area is very remarkable. We witness the transformation of wooded areas other times into other classes of anthropized occupations. These dynamics reveal that the current human pressures on woody plant resources are undoubtedly breaking the self-regenerative capacities of these natural plant formations which are seriously threatened. In addition, there is a rapid expansion of agricultural areas to the detriment of woodland formations. It is therefore more urgent to develop an integrated management strategy at both local and regional levels to preserve these resources and ensure their conservation for sustainable use.

## Data Availability

The data used for this article was collected in the field by the authors and is available and part of the data comes from satellite data.
